# Modulation of resting macrophage activity via low-level laser therapy (LLLT) and α-lipoic acid: an in vitro study using a PCL-based biomaterial

**DOI:** 10.1038/s41598-026-43877-y

**Published:** 2026-03-18

**Authors:** Anna Ścisłowska-Czarnecka, Aleksandra Matuła, Ewa Stodolak-Zych, Amelia Lizak, Joanna Homa, Beata Stenka, Magdalena Chadzinska, Aneta Bac

**Affiliations:** 1 Department of Applied Cosmetology, University of Physical Culture, Al. Jana Pawła II 78, Kraków, 31-571 Poland; 2 Faculty of Materials Science and Ceramics, AGH University of Science and Technology, Kraków, Poland; 3 Department of Evolutionary Immunology, Institute of Zoology and Biomedical Research, Jagiellonian University, Kraków, Poland; 4 Department of Applied Cosmetology, University of Physical Education and Sport, Gdańsk, Poland; 5 Faculty of Motor Rehabilitation, University of Physical Culture, Kraków, Poland

**Keywords:** Resting macrophages, LLLT, PCL poly(ε-caprolactone), α-lipoic acid (LA), Viability, Secretory activity, Biotechnology, Cell biology, Medical research

## Abstract

Modulation of macrophage function represents a promising strategy for promoting tissue remodelling and repair. In the present study the combined effect of low-level laser therapy (LLLT) and α-lipoic acid (LA) on resting RAW 264.7 macrophages cultured in vitro was evaluated. Cells were cultured on LA-modified poly(ε-caprolactone) (PCL) membranes, designed as a controlled release platform. Physicochemical analysis confirmed that LA incorporation decreased PCL crystallinity while increasing membrane surface porosity, providing a biphasic LA release profile. LLLT (808 nm, 200 mW, 5 J/cm², pulsed mode) did not affect macrophage viability but modulated matrix metalloproteinase (MMP-9 and 2) activity and macrophage oxidative-antioxidant potential. Cell culturing carried out on LA-modified membranes (PCL-LA) increased their viability, reduced AK and NO levels as well as TNF-α secretion. Simultaneous treatment with LLLT and LA (PCL-LA-IR) further increased macrophage viability, decreased level of MCP-1/CCL2 chemokine, and modified the presence of pro-MMP-9 as well as the activity of MMP-2, MMP-9 and MMP-9 dimers. Moreover, LA-treated cells showed an increase in antioxidant capacity without oxidative potential being altered. These results indicate that PCL is a stable and effective carrier of LA, and that the observed combined effect of LLLT and LA influences the activity of resting macrophages, which may promote the creation of a favourable environment for regenerative processes. These results also indicate the potential for combined therapies using immunomodulatory biomaterials that support tissue repair to be further developed.

##  Introduction

Present in all tissues resting macrophages maintain homeostasis and support tissue remodelling, and in the event of injury or infection, they initiate cell recruitment to the damage site^1^. Due to their ability to respond to tissue signals, they can differentiate into either the M1 or M2 phenotypes. M1 macrophages are characterised by a proinflammatory profile, secreting, among others, proinflammatory cytokines: TNF-α and IL-1β. In turn, M2 macrophages show anti-inflammatory and regenerative functions, producing anti-inflammatory cytokines, i.e. IL-10 and TGF-β, as well as extracellular matrix remodelling enzymes, including MMP-2 and MMP-9^2,3^.

Proper macrophage functioning is crucial for maintaining balance between the body’s defence response and tissue regeneration. In literature data, it is indicated that macrophage dysregulation, including abnormal transitions between the resting and activated states, promotes chronic inflammation and impaired tissue regeneration^3–5^. Therefore, therapeutic strategies aimed at modulating macrophage activity to support favourable cellular responses and stimulate regenerative processes are gaining in significance^6^. One of such strategies is use of low-level laser therapy (LLLT). LLLT is a physical method belonging to a group of interactions known as photobiomodulation (PBM), which uses low-energy light to modulate cellular processes related to tissue regeneration, including cell proliferation and migration, as well as the secretion of pro-regenerative and angiogenic factors^7–12^. The photobiomodulation efficacy of LLLT depends greatly on the irradiation parameters, such as radiation wavelength, power, energy dose and method of radiation beam application. The wavelength largely determines both the mechanism of light-cell interaction and the nature of the induced biological response^7,13^. It has been shown in numerous in vitro studies that near-infrared radiation is useful in modulating mitochondrial activity and cell repair processes^7,8,13^. In this context, the 808 nm wavelength is of particular interest, as a consistent profile of pro-regenerative effects was observed in various in vitro cell models. For example, Noorizadeh et al. demonstrated that LLLT at the 808 nm wavelength caused changes in the profile of exosomes secreted by cells, indicating their significant role in intercellular communication and the regulation of regenerative processes^14^. Others observed that the use of the same wavelength led to increased viability and enhanced differentiation of stromal cells, and in the case of keratinocytes, to increased migration and induce the phenotypic changes promoting reepithelialization^15,16^. Similarly, Ahmadi et al. demonstrated that the use of the 808 nm wavelength at appropriately selected energy doses increased stem cell proliferation without inducing cytotoxic effects^17^.

A growing body of evidence indicates that the beneficial effects of 808 nm radiation are not limited to structural cells. They also extend to immune cells, particularly macrophages, which play a key role in regulating the transition from the inflammatory to the proliferative phase of the healing process^18^. Previous, in vitro studies found that LLLT at a wavelength of 808 nm enables parameter-dependent modulation of macrophage activity without inducing an excessive proinflammatory response. Golovynska et al. demonstrated that exposure to 808 nm radiation led to a transient increase in phagocytic capacity and adhesion of macrophages, increased mitochondrial activity and mitochondrial membrane potential, while simultaneously reducing the secretion of proinflammatory cytokines (TNF-α, IL-6) and increasing the level of anti-inflammatory cytokines (IL-10, TGF-β)^19^. Similar results were obtained by da Fonseca et al., who demonstrated that 808 nm laser irradiation reduced the secretion of IL-6 and TNF-α without affecting cell viability and nitric oxide production^20^. Silva et al. also confirmed that exposure of macrophages to radiation at the same wavelength did not lead to significant changes in nitric oxide (NO) production across a wide range of tested powers and energy doses^21^.

The data presented from literature on the subject suggest that 808 nm radiation may enable controlled modulation of cell activity, favouring the suppression of the inflammatory response in macrophages and creating conditions that support tissue regeneration. It may be assumed that the observed cellular response profile is related to the interaction of near-infrared radiation with mitochondrial chromophores, in particular cytochrome c oxidase, which potentially leads to changes in mitochondrial activity as well as redox signaling, and consequently, to modulation of inflammatory mediator and pro-regenerative factor secretion^7^.

Also our previous observations regarding the response of resting macrophages from the RAW 264.7 cell line to LLLT at a wavelength of 808 nm demonstrated that 808 nm radiation in pulsed mode at a power of 200 mW and a dose of 5 J/cm² induced the most favourable, anti-inflammatory profile of the cellular response. These conditions led to increased viability and adhesion of macrophages, did not exhibit cytotoxic effects and promoted modulation of their secretory activity, including regulation of nitric oxide production, reduced secretion of proinflammatory cytokines and decreased MMP-9 activity^22^. On this basis, these parameters were assumed to be optimized in the dose-response analysis, enabling the assessment of the immunomodulatory and pro-regenerative effects of LLLT in the applied experimental model.

Similarly to LLLT, biologically active compounds can modulate cellular processes, and their simultaneous use can induce synergistic effects. A valuable source of such compounds are plant extracts containing, *inter alia*, flavonoids, phenols and organic acids with anti-inflammatory and antioxidant properties^23,24^. Among them, α-lipoic acid (LA) a coenzyme involved in metabolic processes and ATP production in cells is particularly noteworthy^25–28^. It exhibits strong antioxidant properties as it neutralises reactive oxygen species, supports the regeneration of glutathione and vitamins C and E, and acts as an important modulator of oxidative stress^26–29^. Moreover, the growing number of studies indicated its immunomodulatory role, including the regulation of macrophage activity and the creation of conditions conducive to tissue regeneration^20–32^. Furthermore, LA supports the transition from the inflammatory phase of the cellular response towards the proliferation process and stimulates angiogenesis, significant in the case of tissue regeneration^33^.

The limited bioavailability and rapid degradation of α-lipoic acid pose a significant therapeutic challenge. As a consequence, methods are being sought to extend its duration of action and prevent loss of biological activity. One promising solution is the use of biodegradable polymer matrices, which not only protect biologically active compounds from enzymatic and oxidative degradation but also enable control of their release rate. Membranes made from polyhydroxy acids, such as PLA, PGLA or PCL, are attracting particular attention, because modifying their composition or structure allows for the appropriate shaping of their properties to function as stable carriers of active substances^34,35^. Biomaterials based on polyhydroxy acids also demonstrate high physicochemical stability and resistance to various physical factors. For example, Zernitckaia et al. showed that the chemical structure of PLA and its PLA/HAP composites remains unchanged after exposure to an electron beam (25 kGy), which confirms their high structural stability^36^. Similar observations were made by Szponder et al., who analysed PCL subjected to a pulsed magnetic and electric field and did not observe any changes in the chemical structure or violation of the integrity of the tested material, which also confirms the high resistance of this polymer to the applied physical stimuli^37^.

Poly(ε-caprolactone) (PCL) was selected both as a biologically relevant in vitro platform and as a potential translational biomaterial for future in vivo applications. PCL is widely described as a biodegradable and biocompatible polymer with good cellular tolerance and has been extensively investigated as a matrix for regenerative applications, including scaffolds and membranes^38–41^. Owing to its favourable physicochemical properties, such as high structural stability, susceptibility to surface and structural modification, and the possibility of functionalization and controlled release of biologically active compounds, PCL constitutes an attractive carrier platform for therapeutically relevant agents^42–44^. These properties support its consideration in regenerative strategies, including advanced wound dressing systems.

Importantly, in the context of tissue regeneration, the interaction between PCL-based biomaterials and immune cells is of particular relevance, as the host immune response can critically modulate inflammation and subsequent repair processes. In previous studies, it has been demonstrated that macrophages effectively adhere to PCL structures, and the topographical as well as structural properties of the material may influence macrophage activation and cytokine secretion profiles^45–47^. Therefore, culturing macrophages directly on PCL membranes provides biologically meaningful information regarding immunomodulatory responses in a clinically relevant material context.

In the present study, the porous morphology of the PCL membrane additionally enabled sustained loading and release of α-lipoic acid, which was essential for investigating the hypothesized synergistic effect of controlled LA delivery and low-level laser therapy (LLLT) on macrophage behaviour. Furthermore, PCL was previously studied in LLLT conditions^44^, allowing us to minimize potential confounding effects of laser irradiation on the polymer itself. Using PCL allowed to investigate macrophage responses in a structurally and chemically defined environment, minimizing variability associated with natural matrices.

Thus, the PCL membrane was not intended solely as a simplified in vitro substrate, but rather as a translationally relevant carrier system enabling the evaluation of combined antioxidant and LLLT effects in a configuration potentially applicable to the development of advanced immunomodulatory wound dressings or implantable regenerative materials.

In the present study, PCL membranes modified with α-lipoic acid were used as an in vitro model to evaluate macrophage responses and provide preliminary characterisation of the LA immunomodulatory properties. This approach allows assessment regarding the biological activity of the system at the biomaterial design stage, prior to further optimization and potential research on applications.

The aim of this study was to evaluate the effects of the combined application of low-level laser therapy (LLLT) and α-lipoic acid (LA) released from PCL membranes on the response of resting macrophages. This was carried out in order to assess the immunomodulatory potential of the system.

## Materials and methods

### Obtaining substrates

Commercial poly(ε-caprolactone) (PCL, 80 kDa, Cat. No. 440744, Sigma-Aldrich, Germany) was used as a carrier for the active compound. The membranes were formed via non-solvent induced phase separation (NIPS) using the THF-DMS-H₂O solvent system (Avantor SA). α-Lipoic acid (α-LA, Cat. No. 437692, Sigma-Aldrich, Germany) was introduced into the polymer solution at 1% w/v prior to the formation process to obtain uniform distribution in the matrix. The membranes were prepared in a precipitation bath (water at 20 °C) and then successively dehydrated in increasing concentrations of ethyl alcohol solutions (50%, 60%, 70% and 96%). The exact procedure for obtaining the membranes is consistent with the protocol described by Szponder et al.^37^.

### Physicochemical characteristics of membranes

Rheological testing (Anthon Paar 1.6 V rotational rheometer) and thermal methods, including thermogravimetry (TG) and differential scanning calorimetry (DSC), were used to evaluate the physicochemical properties of LA-modified PCL membranes. The obtained results allowed to determine the effect of α-lipoic acid on the structural properties and thermal stability of the membranes. The trials were performed in a protective nitrogen atmosphere at temperatures ranging from 10 to 400 °C (Netsch).

### Analysis of membrane morphology

The morphology of the membrane surfaces and interiors was evaluated using scanning electron microscopy (SEM) and porometry. Analysis of pore size distribution revealed a predominance of surface porosity over volume porosity, which may influence the mechanisms of active compound release.

###  α-lipoic acid (LA) release testing

In order to assess the potential for α-lipoic acid release from the polymer matrix, in vitro tests were performed. The membranes were incubated in a PBS buffer (pH 7.4) at 37 °C. Changes in the concentration of released acid in the medium were monitored by turbidimetry at specific time intervals (days 1–7). The α-lipoic acid release profile was monitored turbidimetrically at a wavelength of 600 nm. The results were expressed in absorbance units (A₆₀₀) and converted to concentrations (µg/mL) based on a standard curve.

### Conditions of macrophage culturing

The RAW 264.7 macrophage cell line (Cat. No. TIB-71^™^, ATCC, USA) was used for the study. The cells were cultured in 75 ml plastic bottles (Cat. No. 707001, Nest SB, USA) in an RPMI 1640 culture medium (Cat. No. BE12-702 F, Lonza, USA), supplemented with 10% FBS (Cat. No. 10270-106, Gibco, USA) and 1% antibiotic solution: penicillin and streptomycin (Cat. No. P4333, Sigma-Aldrich, Germany), in a 5% CO_2_ atmosphere at 37 °C. Then, 1 ml of the cell suspension, at a concentration of 1.5 × 10⁴ cells/ml, was placed in a 24-well culture plate (Cat. No. 702001, Nest SB, USA) at the bottom of which reference PCL membranes (PCL group) or PCL membranes modified with α-lipoic acid (PCL-LA group) had been previously placed. Macrophages exhibited spontaneous adhesion to the membrane surface during culture. The culture medium was not changed throughout the experiment to ensure consistent culture conditions and allow for the accumulation of factors secreted by the cells into the medium throughout the duration of the experiment.

### Low-level laser therapy (LLLT)

Laser irradiation of macrophages cultured on PCL membranes (PCL-IR group) and on α-lipoic acid**-**modified PCL membranes (PCL**-**LA**-**IR group) was performed using low-level laser therapy (LLLT). Irradiation was carried out with the PhysioGo 400 C laser device (A-UL-AST-PHG400C, ASTAR, Poland). This is a low-energy laser that generates electromagnetic radiation in the infrared range with a wavelength of 808 nm, a power of 200 mW. The irradiation time per single session was 49 s and a radiation dose of 5 J/cm^2^/cell well. The laser beam spot size was 10 mm in diameter. The laser beam was applied in a pulsed (I) manner (frequency of 100 Hz, with a 50% duty cycle). Irradiation of the surface was performed using a non-contact method at a minimum distance of 1 cm from the cells (the height of the well) and at a 90*°* angle. Laser radiation was applied every 24 h, 2, 4 and 6 times. On the following days of the experiment: day 3 (2 laser beam applications), day 5 (4 laser beam applications) and day 7 (6 laser beam applications), the macrophage culture was terminated and the cells as well as the supernatant collected from the culture were used for further biological assays.

### Cell viability (ViaLight test)

To test macrophage viability during culturing, a ready-made assay with the ViaLight reagent kit (Cat. No. LT07-221, Lozna, Switzerland) was used. A cell lysis reagent in the amount of 200 µl was added to wells containing cells and 600 µl of a supernatant. Following 10 min of incubation, 200 µl of the supernatant-lyser mixture was transferred to a white 96-well plate (Cat. No. 655083, Nest SB, USA), and 200 µl of the AMR PLUS reagent was added. After 2 min, the amount of emitted irradiation was determined using the FLUOstar Omega reader (BMG Labtech, Germany).

### Level of released adenylate kinase AK (ToxiLight test)

Adenylate kinase (AK) levels were determined via bioluminescence quantification using the Toxilight reagent kit (Cat. No. LT07-217, Lonza, Switzerland). The cell culture supernatant in the amount of 20 µl was collected and transferred to a white 96-well plate (Cat. No. 655083, Nest SB, USA). Then, 100 µl of the AK Detection Reagent solution was added to each well. After 5 min of incubation, luminescence was read using the FLUOstar Omega reader (BMG Labtech, Germany).

### Level of released nitric oxide NO (Griess test)

Into each well of the transparent 96-well plate (Cat. No. 701001, Nest SB, USA),100 µl of a cell supernatant was transferred, and 100 µl of the reagent mixture (Sigma-Aldrich, Germany) was added - Griess A (1% sulphanilamide, Cat. No. S9251, Sigma-Aldrich, Germany) in 5% phosphate acid) and B (0.1% (naphthyl)enediamine, Cat. No. N9125, Sigma-Aldrich, Germany) in H_2_O mixed at a 1:1 ratio. After 5 min, the optical density (O.D.) of the fluid was read at 540 nm using the FLUOstar Omega reader (BMG Labtech, Germany).

### Level of secreted cytokines

Cytokine levels in the cell culture supernatants were measured via flow cytometry using Flex Set kits (CBA, BD Biosciences). The entire assay procedure, measurements and analyses were performed according to the instructions included in the cytokine assay kit using the Beckman Coulter flow cytometer (Life Science, USA). The Mouse Inflammation Kit (Cat. No. 552364BD Biosciences, USA) was used, which allows for simultaneous determination of 6 cytokine levels: tumour necrosis factor (TNF-α), interleukin 6 (IL-6), interleukin 10 (IL-10), monocyte chemoattractant protein-1(MCP-1), interferon gamma (IFN-γ) and interleukin 12p70 (IL-12p70) (BD Biosciences, USA; Cat. No. 51-9005809; 51-9005811; 51-9005813; 51-9005815; 51-9005807; 51-9005817). Data analyses and determination of cytokine concentrations were performed in Microsoft Excel using standard curves, which were prepared based on successive dilutions of the standard. The detailed procedure has been previously described^22^.

### Level of secreted metalloproteinases

The level of matrix metalloproteinases secreted by the cells, both in the form of inactive proenzymes and active forms, was measured using gelatine zymography. This is a modified electrophoretic method that allows for the assessment of proteolytic enzyme activity, the substrate of which (gelatine) is incorporated into a polyacrylamide gel with the addition of SDS (sodium dodecyl sulfate). This method allows for the differentiation and determination of pro-MMP-2, pro-MMP-9, as well as the active forms of MMP-2 and MMP-9. A detailed zymography protocol has been previously described^22^.

### Measurement of oxidation potential (PerOx test - TOS/TOC)

Total oxidative status (TOS) of the cells was determined by measuring lipid peroxide levels according to the instructions for use of the PerOx (TOS/TOC) kit (Cat. nNo. KC5100, Immunodiagnostik AG, Bensheim, Germany). Peroxide levels in the tested cell culture supernatant samples were determined by reacting horseradish peroxidase with tetramethylbenzidine dichloride (TMB) in the presence of hydrogen peroxide. The reaction with the enzyme produces a soluble blue product. It was stopped by adding 2 M H_2_SO_4_, which led to a colour change from blue to yellow^22^.

### Measurement of antioxidant potential (ImAnOx test - TAS/TAC)

Total antioxidant status (TAS) of the cells was determined by reacting antioxidants with a predetermined (known) amount of exogenous hydrogen peroxide (H_2_O_2_), according to the ImAnOx (TAS/TAC) kit protocol (Cat. No. KC5200, Immunodiagnostik AG, Bensheim, Germany). In this assay, antioxidants react with peroxide, and the amount of unreacted H_2_O_2_ is measured spectrophotometrically. The difference between the added and measured amounts of H_2_O_2_ (relative to the calibrator included in the kit) is proportional to the antioxidant activity^22^.

### Statistical analysis

Statistical analysis was performed after verifying that the parametric tests were met. Normality of data distribution was assessed using the Shapiro-Wilk test. Normally distributed data were analysed using the Student’s *t*-test for independent samples, while data that did not met the assumptions of normality were analysed using the non-parametric Mann-Whitney U test. The results are presented as means ± standard deviations. *P* values ​​< 0.05 were considered statistically significant. All statistical analyses were performed as two-group comparisons (PCL vs. PCL-IR; PCL vs. PCL-LA; and PCL-LA vs. PCL-LA-IR), separately for days 3, 5, and 7 of culture.

## Results

###  Characteristics of substrates modified with α-lipoic acid (LA)

**Fig. 1:**
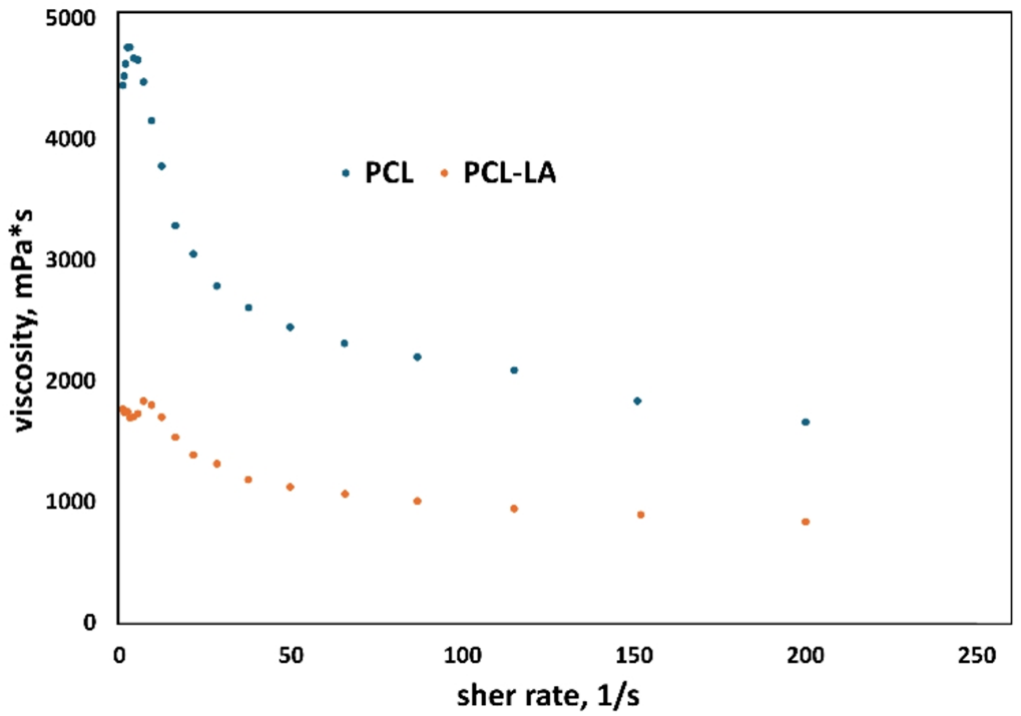
Viscosity curves of membrane-forming solution for polycaprolactone polymer with added α-lipoic acid (PCL-LA) and unmodified polycaprolactone solution (PCL).

The addition of α-lipoic acid clearly affects the rheological properties of the polymer solution. Across the entire shear rate range, the PCL-LA sample exhibited higher viscosity values ​​compared to pure PCL, which may indicate the presence of interactions between LA molecules and polymer chains (Fig. 1). At the same time, both systems demonstrate non-Newtonian shear-thinning behaviour, typical of polymer solutions formed by the phase inversion method. The increase in viscosity after the addition of LA may suggest that this compound, despite its partially amphiphilic nature, does not act as a classic surfactant lowering interfacial tension, but rather stabilises the system by forming additional or hydrogen bonds with PCL. This change in rheology may affect the demixification process during membrane formation, as reflected in the increased surface porosity and more heterogeneous microstructure observed in the SEM image (Fig. 1).

The addition of 1% (w/w) α-lipoic acid to the PCL matrix caused a significant decrease in the enthalpy of melting (Hm) from 70.14 J/g to 54.24 J/g, and a decrease in the determined crystallinity from 44.7% to 34.6%. The melting temperature (Tm) remained practically unchanged (60.5 → 61.2 °C), while the glass transition temperature (Tg) increased from 30.2 °C to 38.2 °C. The degradation temperature (Td) did not change significantly (≈ 415 °C), indicating preserved thermal stability of the material. The decrease in crystallinity indicates an increase in the amorphous fraction of the PCL-LA sample, which usually favours diffusion and faster release of the active substance. On the other hand, a significant increase in Tg suggests limitation of mobility regarding polymer chain segments (probably due to interactions between LA and PCL), which may inhibit diffusion at the temperature of incubation (37 °C) (Table 1). Therefore, the effect of LA addition on the release kinetics is the result of a competition between the increased availability of amorphous diffusion paths and the reduced segmental mobility of the matrix.

**Table 1:** Comparison of characteristic temperatures and parameters determined on their basis for polycaprolactone reference PCL membrane (PCL) and polycaprolactone membranes PCL modified with α-lipoic acid (PCL-LA).

	Hm[J/g]	Crystallinity[%]	Tg[C]	Tm[C]	Td[C]
Reference membrane, PCL	70.14	44.68	30.2	60.5	414.8
Membrane, PCL-LA	54.24	34.55	38.2	61.2	415.8

### Morphology of membrane surfaces

**Fig. 2:**
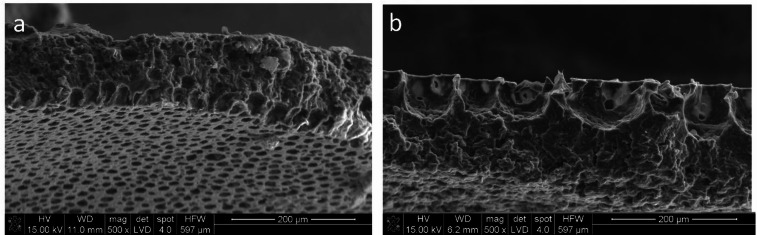
Morphology of polycaprolactone PCL**-**based membranes: a)polycaprolactone membranes PCL modified with lipoic acid (PCL-LA) and b) polycaprolactone reference membrane (PCL).

SEM observations (Figs. 2a, b) indicate that the incorporation of 1% (w/v) α-lipoic acid into the PCL matrix (PCL-LA) leads to increased surface and volume porosity. Furthermore, the volume porosity is less uniform, with pores predominating in the 30–40 μm range. The LA-containing membrane is characterised by a higher proportion of open pores and irregular morphology, suggesting that the presence of the active compound influences the phase separation process during membrane formation. Increased surface porosity may favour faster α-lipoic acid release at the initial stage of incubation, while the heterogeneous structure of the membrane interior may determine the long-term nature of diffusion in the later release phase.

SEM analysis of the PCL and PCL-LA membranes revealed a porous structure with two dominant pore populations. Surface pores had an average diameter of 55 μm (median 40–58 μm, 48% of total pores), while pores within the membrane bulk averaged 26 μm (median 25–30 μm, 67% of total pores). The overall porosity of the PCL membrane, considering both surface and bulk pores, was approximately 52%. Incorporation of α-lipoic acid (LA) into the PCL matrix did not significantly alter the microstructure. PCL-LA membranes still exhibited two primary pore populations, with surface pores averaging 56 μm (median 40–58 μm) and bulk pores averaging 28 μm (median 2530 μm). Total porosity of PCL-LA membranes remained similar at 51%. These results indicate that the addition of LA does not substantially modify the membrane architecture, suggesting that any observed biological effects are likely due to the release of LA rather than structural changes in the PCL matrix.

###  α-lipoic acid (LA) release

**Fig. 3:**
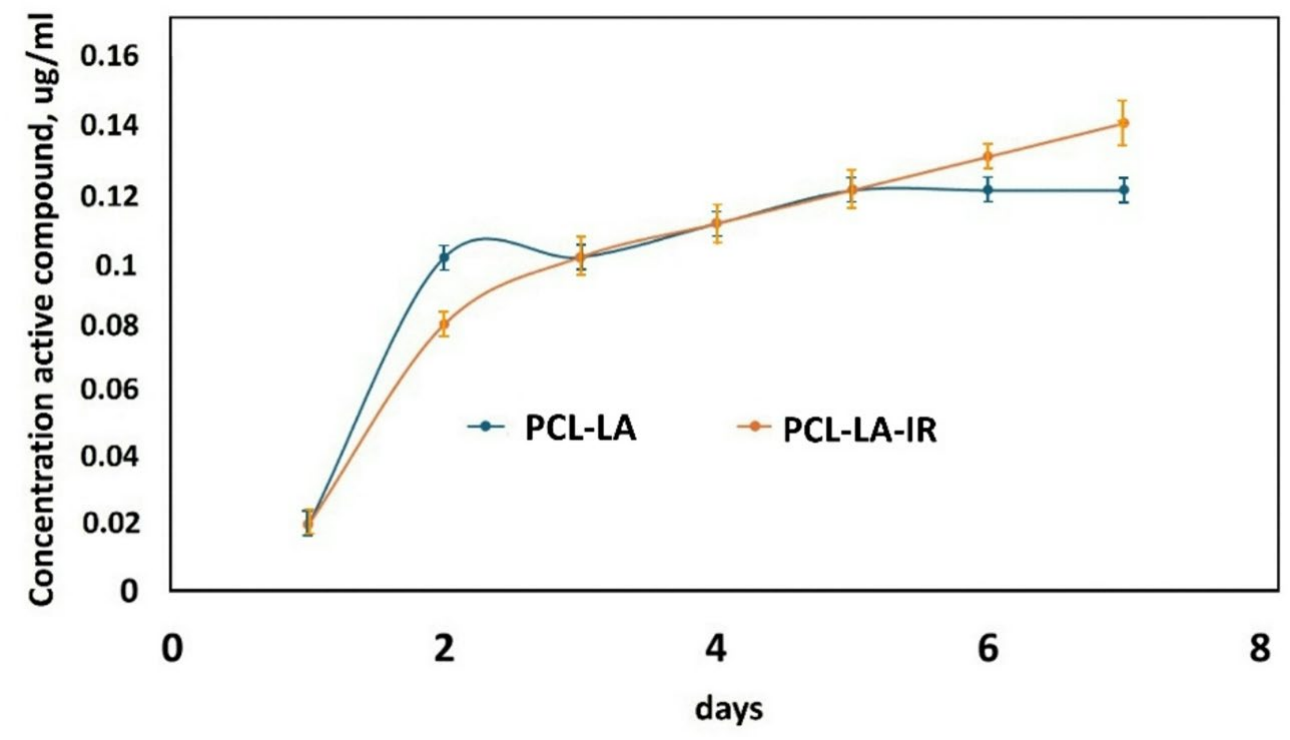
Release curve of α-lipoic acid from polycaprolactone membranes PCL modified with α**-**lipoic acid (PCL-LA) and polycaprolactone membranes PCL modified with α**-**lipoic acid and irradiated with LLLT (PCL-LA-IR).

Turbidimetric tests (PBS, 37 °C) demonstrated a characteristic, two-phase process of LA release from the PCL membrane. In the first phase (up to 48 h), a rapid increase concerning the compound concentration in the medium was observed, corresponding to the release of molecules from the porous membrane surface. Subsequently, the release rate slowed down significantly and reached a plateau (Fig. 3). The obtained profile correlates with previous SEM observations, which indicate a predominance of surface porosity over volumetric porosity, and with DSC results, where an increase in Tg and a decrease in crystallinity suggest limited mobility of polymer chains. The high viscosity of the PCL-LA solution confirms the existence of interactions between LA and PCL, which may stabilise the matrix and hinder further diffusion of molecules. As a result, the LA release process occurs primarily from the surface and is self-limiting, which may contribute to the effect of prolonged biological action.

###  Viability and secretory activity of macrophages cultured on PCL membranes and exposed to low-level laser therapy

#### Cell viability and secretory activity (AK and NO)

**Fig. 4:**
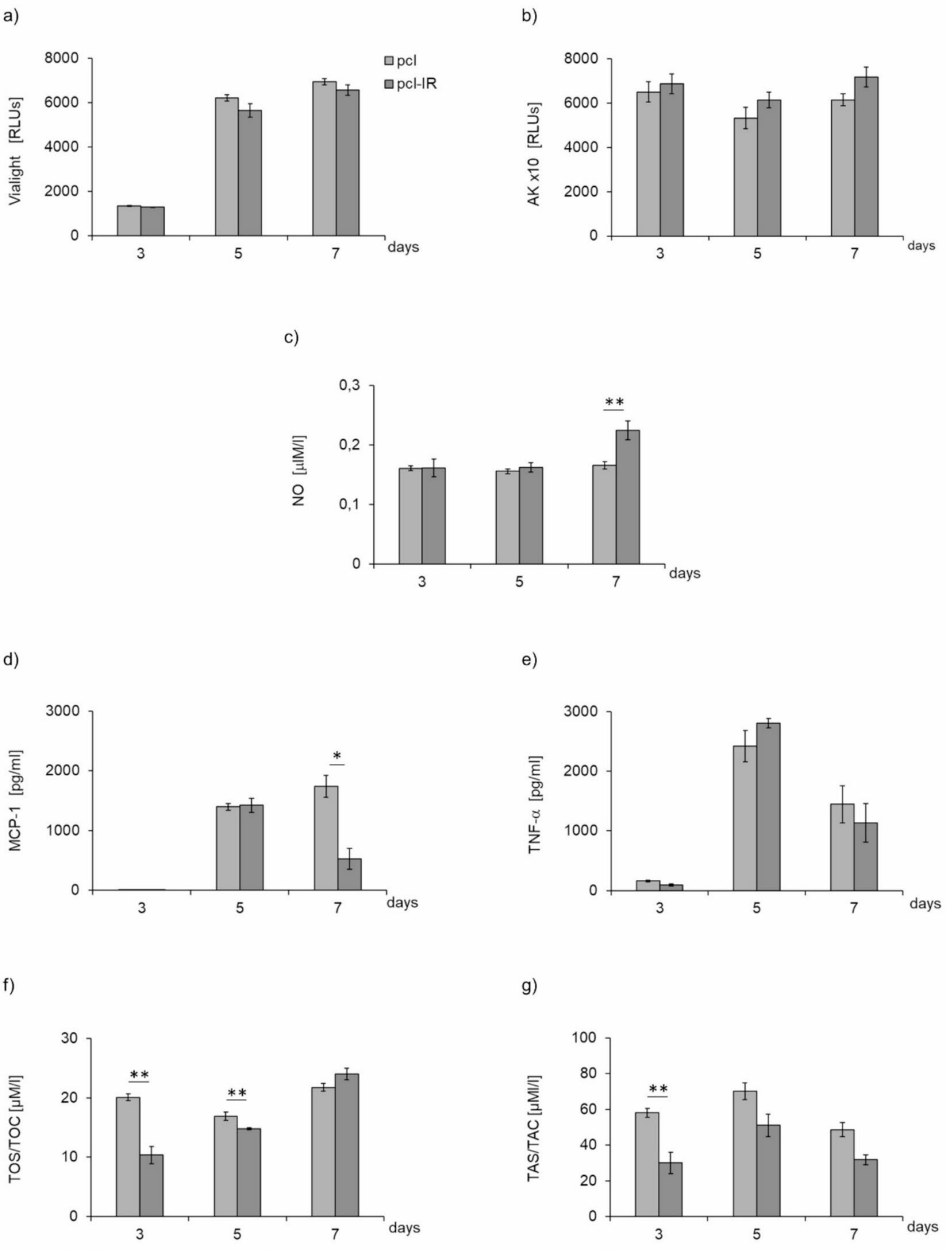
Effect of porous polycaprolactone membrane (PCL) and pulsed laser beam irradiation on viability (**a**) and levels of released adenylate kinase (AK), (**b**), secreted nitric oxide (NO), (**c**), MCP-1/CCL2 (4d), TNF-α (**e**), oxidative (TOS/TOC, 4f) and antioxidant (TAS/TAC, 4 g) potential of RAW 264.7 cell line macrophages. Cells were cultured on PCL membranes (PCL) and cultured on PCL membranes and irradiated (PCL-IR) with laser at powers of 200 mW and radiation doses of 5 J/cm^2^/well with cells (frequency 100 Hz, 50% duty cycle) at wavelength of 808 nm. Parameters were measured on consecutive days (3, 5 and 7) of the experiment. RLUs **-** luminometer flux unit. Viability and adenylate kinase (AK) results are presented as means ± SE from three independent experiments (*n* = 3). The remaining parameters are shown as means ± SE calculated from 2–3 samples per experimental repetition. *, ** **-** differences between cells irradiated with laser (PCL-IR) and cells non-irradiated (PCL), (* *p* < 0.05, ** *p* < 0.01).

Irradiation of macrophages in the PCL-IR group did not affect their viability and the level of adenylate kinase (AK) release at any of the examined time points (days 3, 5 and 7 of culture) (Figs. 4a, b, respectively), which indicates the absence of a detectable cytotoxic effect in the applied experimental conditions. On day 7, the group of irradiated cells (PCL-IR group) showed a pronounced increased secretion of nitric oxide (NO) secretion compared to the control cells (PCL group) (Fig. 4c). The delayed increase in NO secretion may reflect a late macrophage response rather than an acute pro-inflammatory activation, as supported by the lack of changes in TNF-α secretion (presented on Fig. 4c, e).

####  Cytokine and chemokine secretion by macrophages

On day 7 of macrophage culture, an approximately 2.5-fold decrease in MCP-1/CCL2 secretion by the irradiated macrophages (PCL-IR group) was observed compared to PCL group, while the level of TNF-α remained unchanged compared to the control group (PCL group) as demonstrated by quantitative analysis (Fig. 4d and e) and confirmed by representative cytokine dot plots obtained by flow cytometry (Fig. 6). The results suggest changes in chemotactic signalling, without overall inhibition of the inflammatory response.

The remaining analysed cytokines: IFN-γ, IL-12p70, IL-6 and IL-10 were not detectable in any of the groups (PCL and PCL-IR) and at any of the examined time points.

####  Matrix metalloproteinase (MMP) activity of macrophages

In the PCL-IR group, an approximately four-fold decrease in MMP-2 activity was observed on day 3 of macrophage culture. Moreover, on day 5 of culture, a sixteen-fold increase, and on day 7, an approximately five-fold increase in gelatinolytic activity corresponding to the pro-MMP-9 band was noted. Moreover, clearly increased MMP-9 activity was detected in supernatants from PCL-IR on days 3 and 5 of culture, followed by a decrease on day 7, compared to the supernatants retrieved from the macrophage of non-irradiated control group (PCL).

Additionally, on day 7 of macrophage culture, a seven-fold increased MMP-9 dimer activity was observed in the PCL-IR group. Such pattern of changes reflects temporally differentiated activity of enzymes involved in extracellular matrix remodelling, including MMP-9, encompassing different molecular forms of this enzyme. These changes are reflected in the quantitative data MMP (Table 2) and visualized in the representative zymograms (Fig. 7).

####  Oxidant-antioxidant status of macrophages

In terms of oxidant-antioxidant balance, a pronounced decrease in oxidative potential (TOS/TOC) was observed in the PCL-IR group on day 3, which persisted on day 5 of culturing compared to the control group (PCL group) (Fig. 4f). However, the antioxidant potential (TAS/TAC) in the PCL-IR group was pronouncedly reduced on day 3 of macrophage culture (Fig. 4g), which reflects the transient modulation of redox homeostasis rather than the induction of oxidative stress.

### Viability and secretory activity of macrophages cultured on PCL membranes modified with α-lipoic acid (LA)

#### Cell viability and secretory activity (AK and NO)

**Fig. 5:**
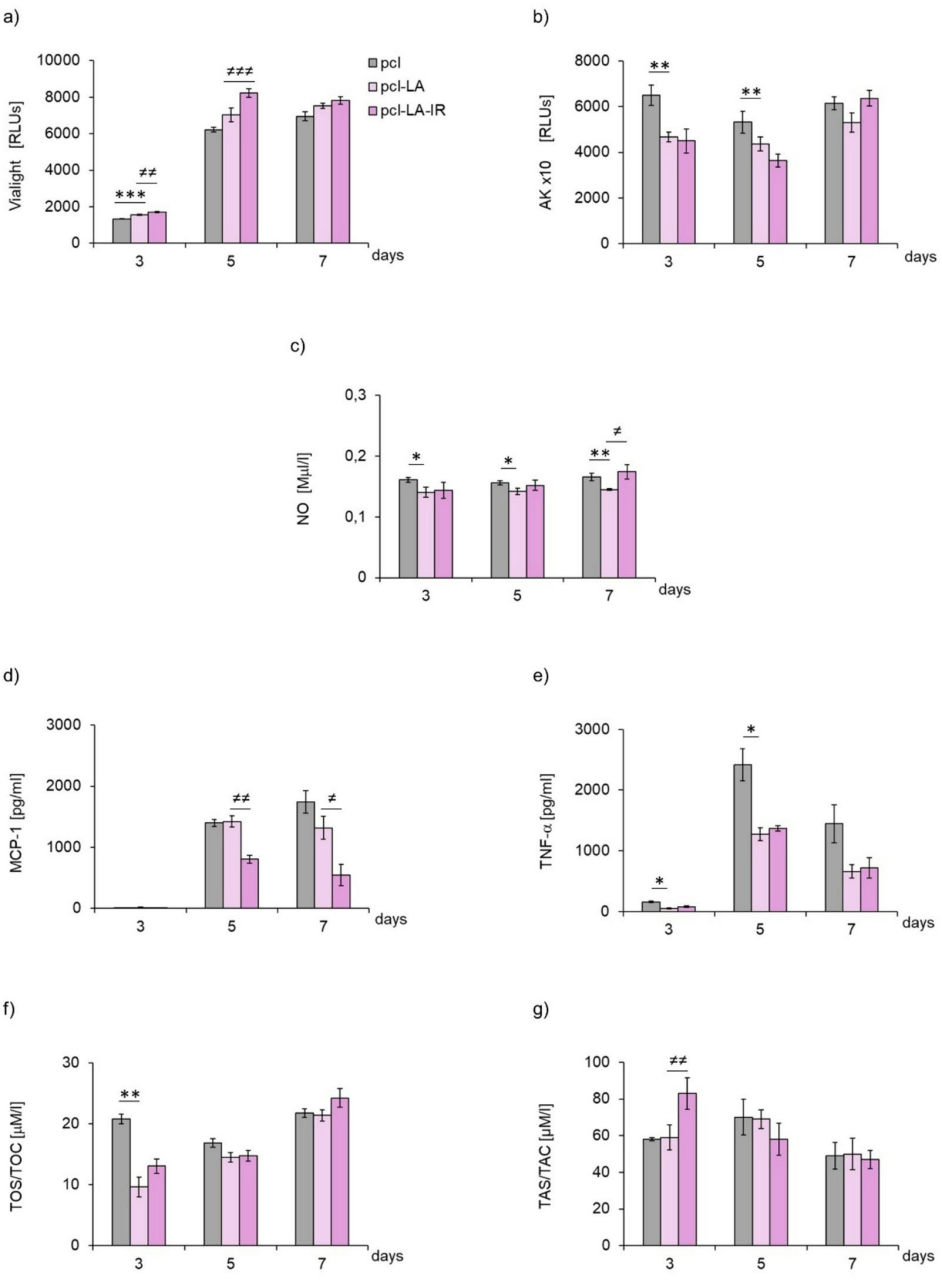
Effect of porous polycaprolactone membrane modified with LA α**-**lipoic acid (PCL-LA) and irradiated with pulsed laser at power of 200 mW and radiation dose of 5 J/cm^2^/well with cells (frequency 100 Hz, 50% duty cycle), at wavelength of 808 nm (PCL-LA-IR) on viability (**a**) and levels of released adenylate kinase (AK)(**b**), secreted nitric oxide (NO, (**c**), MCP-1/CCL-2 (**d**), TNF-α (**e**), oxidative (TOS/TOC, (**f**) and antioxidant (TAS/TAC, 5 g) potential of RAW 264.7 cell line macrophages. Cells were cultured on polycaprolactone membranes PCL (PCL), polycaprolactone membranes modified with α**-**lipoic acid (PCL-LA) and polycaprolactone membranes modified with α**-**lipoic acid and irradiated (PCL-LA-IR). Parameters were measured on consecutive days (3, 5 and 7) of the experiment. RLUs **-** luminometer flux unit. Viability and adenylate kinase (AK) results are presented as means ± SE from three independent experiments (*n* = 3). The remaining parameters are shown as means ± SE calculated from 2–3 samples per experimental repetition. Differences (* *p* < 0.05, ** *p* < 0.01, *** *p* < 0.001) between cells cultured on polycaprolactone membranes modified with α**-**lipoic acid (PCL-LA) and reference PCL group (PCL vs. PCL-LA). Differences (^≠^ *p* < 0.05, ^≠ ≠^ *p* < 0.01, ^≠ ≠^ *p* < 0.001) between cells cultured on polycaprolactone membranes modified with lipoic acid non-irradiated and cultured on polycaprolactone membranes modified with α**-**lipoic acid and cultured on polycaprolactone membranes modified with α**-**lipoic acid and irradiated with laser beam (PCL-LA vs. PCL-LA-IR).

Culturing macrophages on the PCL material modified with α-lipoic acid (PCL-LA group) increased cell viability on day 3 compared to the control cells cultured on PCL (PCL group), while leading to a pronounced decrease in the level of AK release on days 3 and 5, and a reduction in NO secretion at all tested time points (days 3, 5 and 7 of culturing) (Figs. 5a, b, c, respectively). The sustained decrease in NO production is consistent with the anti-inflammatory and redox-modulating properties of α-lipoic acid.

#### Cytokine and chemokine secretion by macrophages

**Fig. 6:**
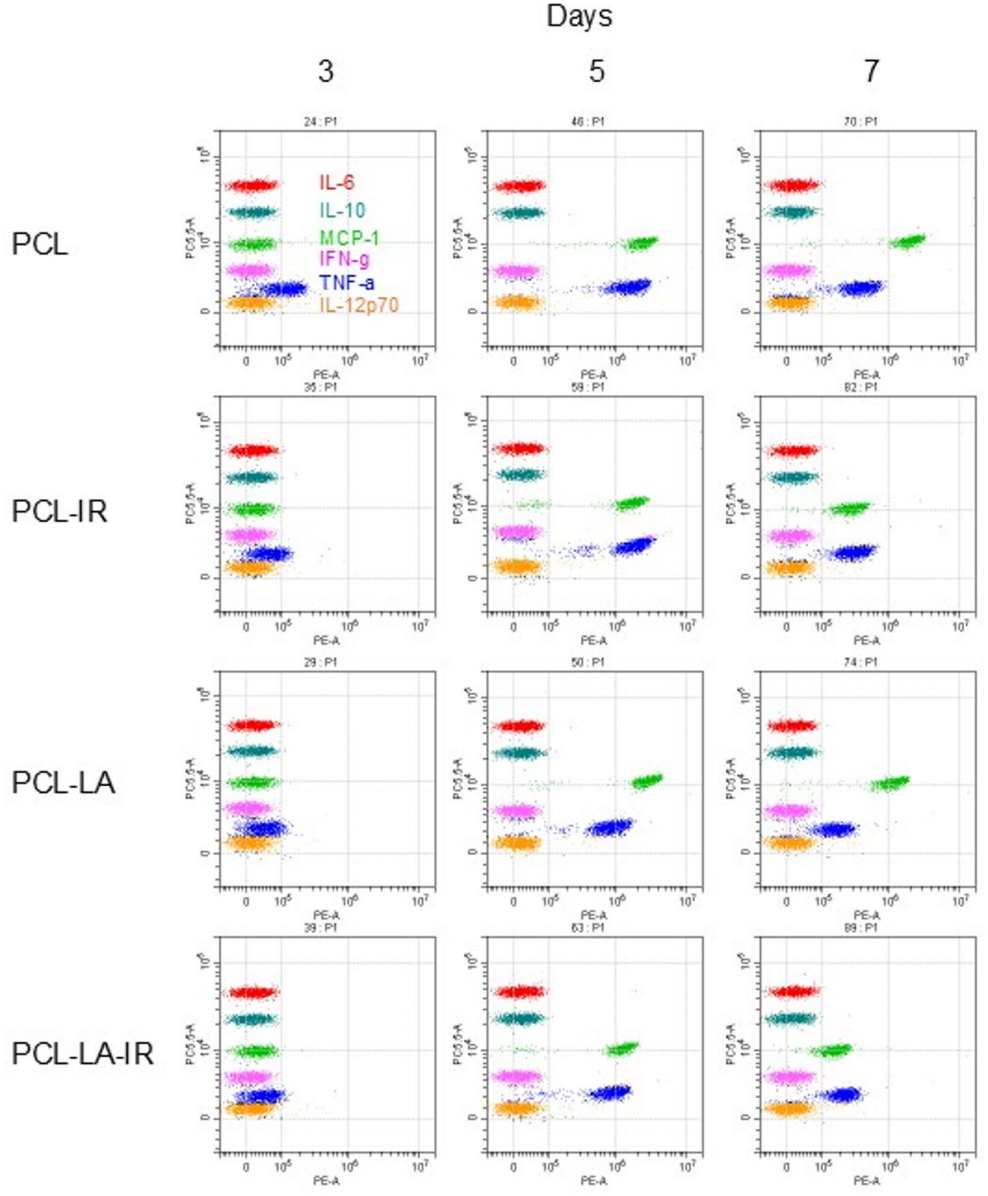
Example/representative dot plots for cytometric analysis of fluorescently labelled cytokines. Cells were cultured for specified number of days on polycaprolactone membranes PCL (PCL), polycaprolactone membranes PCL and irradiated (PCL-IR), polycaprolactone membranes modified with α-lipoic acid (PCL-LA) and polycaprolactone membranes modified with α-lipoic acid and irradiated (PCL-LA-IR) with laser at power of 200 mW and radiation dose of 5 J/cm^2^/well with cells (frequency 100 Hz, 50% duty cycle), at wavelength of 808 nm (groups PCL-IR, PCL-LA-IR). On days 3, 5 and 7 of the experiment, cytokine (TNF-α, IL-6, IL-10, MCP-1, IFN-γ, IL-12p70) levels were determined. Cytokine dot blot analysis was performed using one representative sample per group from each of three independent experiments (*n* = 3).

On days 3, 5 and 7 of culturing, the level of MCP-1/CCL-2 remained unchanged in the group of macrophages cultured on α-lipoic acid-modified PCL (PCL-LA group) compared to the control cells (PCL group), as demonstrated by quantitative analysis (Fig. 5d) and representative cytokine dot plots (Fig. 6). At the same time, TNF-α secretion in macrophages cultured on PCL-LA (PCL-LA group) was reduced approximately three times on day 3 and nearly two times on day 5 compared to the control cells (PCL group), highlighting the differential nature of the observed changes in MCP-1/CCL-2 and TNF-α secretion, as demonstrated by quantitative analysis (Fig. 5e) and representative cytokine dot plots (Fig. 6). The IFN-γ, IL-12p70, IL-6 and IL-10 cytokines were not detected in any of the groups (PCL and PCL-LA groups), regardless of the examination time point.

####  Matrix metalloproteinase (MMP) activity of macrophages

**Fig. 7:**
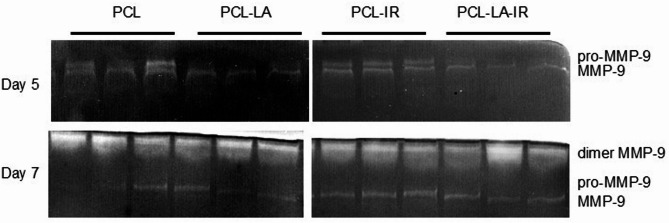
Representative gelatine zymogram of the RAW 264.7 cell line macrophage supernatant. Cells were cultured for specified number of days on polycaprolactone membranes (PCL) and polycaprolactone membranes modified with α-lipoic acid (PCL-LA) and polycaprolactone membranes modified with α-lipoic acid and irradiated (PCL-LA-IR) with laser at power of 200 mW and radiation dose of 5 J/cm^2^/well with cells (frequency 100 Hz, 50% duty cycle), at wavelength of 808 nm (groups PCL-IR, PCL-LA-IR). MMP were determined on consecutive days (3, 5 and 7) of the experiment. Images showing results for individual experimental groups (PCL and PCL/LA and PCL-IR and PCL-LA-IR) were obtained from the same gel. The gel was cropped for clarity; all lanes originate from a single gel run. Full-length, uncropped gel images are provided in the ‘Supplementary Information’. Representative gelatine zymograms were obtained using one representative sample per group from each of three independent experiments (*n* = 3).

Modification of PCL with α-lipoic acid did not affect MMP-2 activity (Table 2). On day 5 of the culture, a decrease in gelatinolytic activity corresponding to the pro-MMP-9 band was observed in the PCL-LA group compared to the control (PCL group). Simultaneously, in the same group (PCL-LA group), an almost six-fold increase in MMP-9 activity was noted on day 5, while its decrease was observed on day 7 of culturing. This transient increase in MMP-9 activity may reflect short-term activation of matrix remodelling processes, followed by their resolution at later stages of cell culture. Changes in pro-MMP-9 and MMP-9 activity are summarised in Table 2 and visualised in the representative gelatine zymograms (Fig. 7).

#### Oxidant-antioxidant status of macrophages

In terms of oxidant-antioxidant parameters, pronounced decreased oxidative potential (TOS/TOC) was observed on day 3 of macrophage culturing on PCL modified with α-lipoic acid (PCL-LA group) compared to the control (PCL) (Fig. 5f). Antioxidant potential (TAS/TAC) did not show significant differences between groups (PCL-LA vs. PCL group) at any of the analysed time points (Fig. 5g), which indicates the absence of sustained changes concerning the total antioxidant potential in the investigated conditions.

### Viability and secretory activity of macrophages cultured on PCL membranes modified with α-lipoic acid (LA) and subjected to low-level laser irradiation

####  Cell viability and secretory activity (AK and NO)

Irradiation of macrophages cultured on PCL modified with α-lipoic acid (PCL-LA-IR group) led to increased cell viability on days 3 and 5 of culturing compared to the non-irradiated group (PCL-LA) (Fig. 5a). At the same time, cell irradiation did not affect the level of AK release in the PCL-LA-IR group and resulted in a slight increase in NO secretion on day 7 of culturing compared to the PCL-LA group (Figs. 5b, c, respectively), which indicates a temporally differentiated response, comprising early changes in cell viability and a later increase in NO levels, with no changes in AK release.

#### Cytokine and chemokine secretion by macrophages

On days 5 and 7 of culturing, a pronounced decrease in MCP-1/CCL-2 secretion was observed in the PCL-LA-IR compared to the PCL-LA group, with no changes in TNF-α levels at any of the analysed time points (days 3, 5 and 7). These findings confirm the selective nature of the observed changes in cytokine secretion, as demonstrated by quantitative analysis (Fig. 5d and e) and representative cytokine dot plots obtained by flow cytometry (Fig. 6). Cytokines IFN-γ, IL-12p70, IL-6 and IL-10 were not detected in any of the studied groups (PCL-LA or PCL-LA-IR), regardless of the examined time point.

#### Matrix metalloproteinase (MMP) activity of macrophages

Irradiation of macrophages cultured on PCL membranes modified with α-lipoic acid (PCL-LA-IR) led to an approximately six-fold reduction in MMP-2 activity on day 3 of culturing, as demonstrated by quantitative densitometric analysis (Table 1) and illustrated by representative gelatine zymograms (Fig. 7). On days 5 and 7, an increase in gelatinolytic activity corresponding to the pro-MMP-9 band was observed, with a particularly pronounced effect on day 7, reaching an approximately six-fold increase. Simultaneously, on day 3, increased activity of the active form of MMP-9 was observed, followed by a pronounced decrease on days 5 and 7 compared to the PCL-LA group, with a decrease exceeding ten-fold evident on day 5 as summarised in Table 2 and visualised in the representative zymograms (Fig. 7). Furthermore, on day 7, an increase in MMP-9 dimer activity was detected in the PCL-LA-IR compared to the PCL-LA group, as shown by quantitative analysis (Table 2) and illustrated in the representative gelatine zymogram (Fig. 7). The observed sequence of changes, involving different forms of MMP-9 at successive time points, suggest coordinated regulation of extracellular matrix remodelling.

#### Oxidant-antioxidant status of macrophages

In terms of oxidant-antioxidant parameters, the oxidative potential (TOS/TOC) in the PCL-LA-IR group did not differ from the PCL-LA group at any of the analysed time points (days 3, 5 and 7) (Fig. 5f). In turn, the antioxidant potential (TAS/TAC) was pronounced higher on day 3 in the irradiated cell group (PCL-LA-IR) compared to group not undergoing irradiation (PCL-LA) (Fig. 5g), which indicates transient changes in antioxidant parameters at an early stage of the experiment.

**Table 2:** Effect of LLLT and α-lipoic acid on MMP activity of RAW 264.7 cell line macrophages.

Parameters	Groups	Day 3	Day 5	Day 7
		$\overline{x}$ ± SD	$\overline{x}$ ± SD	$\overline{x}$ ± SD
MMP-2	PCL	4336.526 ± 1127.712	nd	nd
	PCL-IR	1110.944 ± 189.360	nd	nd
	PCL-LA	3207.784 ± 977.906	nd	nd
	PCL-LA-IR	478.872 ± 64 680	nd	nd
Between group comparison		PCL vs PCL-IR *p* = 0.0476*	-	-
		PCL vs PCL-LA *p* = 0.4911		
		PCL-LA vs PCL-LA-IR *p* = 0.0494*		
Pro-MMP-9	PCL	nd	1988.917 ± 352.929	3749.960 ± 2081.441
	PCL-IR	nd	31,912.684 ± 3956.213	21,261.271 ± 1515.807
	PCL-LA	nd	nd	3553.012 ± 795.533
	PCL-LA-IR	nd	2677.108 ± 615.369	21,384.573 ± 5061.735
Between group comparison			PCL vs PCL-IR *p* = 0.0043**	PCL vs PCL-IR *p* = 0.0062**
			PCL vs PCL-LA *p* = 0.0119*	PCL vs PCL-LA *p* = 0.9485
			PCL-LA vs PCL-LA-IR *p* = 0.0278*	PCL-LA vs PCL-LA-IR *p* = 0.0539*
MMP-9	PCL	nd	7093.060 2227.770	1862.370 643.841
	PCL-IR	1221.077 ± 82.065	15,458.227 ± 1982.039	nd
	PCL-LA	nd	41,423.757 ± 4325.101	920.000 44.753
	PCL-LA-IR	271.370 48.133	2811.970 ± 501.250	nd
Between group comparison		PCL vs PCL-IR *p* = 0.0001***	PCL vs PCL-IR *p* = 0.0483*	PCL vs PCL-IR *p* = 0.011**
		PCL vs PCL-LA *p* = −	PCL vs PCL-LA *p* = 0.0078**	PCL vs PCL-LA *p* = 0.0020**
		PCL-LA vs PCL-LA-IR *p* = 0.0127*	PCL-LA vs PCL-LA-IR *p* = 0.0024**	PCL-LA vs PCL-LA-IR *p* = 0.0001***
Dimer MMP-9	PCL	nd	nd	66,393 6194.579
	PCL-IR	nd	nd	473,806.000 78,599.148
	PCL-LA	nd	nd	56,022.113 3399.013
	PCL-LA-IR	nd	nd	214,453.683 11,676.216
Between group comparison				PCL vs PCL-IR *p* = 0.0159*
				PCL vs PCL-LA *p* = 0.3179
				PCL-LA vs PCL-LA-IR *p* = 0.0005***

Cells were cultured for a specified number of days on polycaprolactone membranes (PCL), polycaprolactone PCL membranes and irradiated (PCL-IR), polycaprolactone membranes modified with α-lipoic acid (PCL-LA), polycaprolactone membranes modified with α-lipoic acid and polycaprolactone membranes modified with α-lipoic acid and irradiated (PCL-LA-IR) with a laser at a power of 200 mW and radiation dose of 5 J/cm^2^/well with cells (frequency 100 Hz, 50% duty cycle), at a wavelength of 808 nm (groups PCL-IR, PCL-LA-IR). Activity of MMP were determined on consecutive days (3, 5 and 7) of the experiment. The average areas of the peaks corresponding to MMP-2, Pro-MMP-9, MMP-9 and Dimer MMP-9 in the samples were calculated and expressed as raw mean values (Raw Vol.). Quantitative analysis was performed using one representative sample per group from each of three independent experiments (*n* = 3) and MMP are presented as mean ± SE; nd (not detected). Differences (* *p* < 0.05, ** *p* < 0.01, *** *p* < 0.001) between cells cultured on polycaprolactone PCL membranes and cells cultured on polycaprolactone PCL membranes and irradiated with laser beam (PCL-IR vs. PCL), differences between cells cultured on polycaprolactone membranes modified with α-lipoic acid (PCL-LA) and reference polycaprolactone membranes PCL (PCL), (PCL-LA vs. PCL), and between cells cultured on polycaprolactone membranes modified with α-lipoic acid (PCL-LA) and polycaprolactone membranes modified with α-lipoic acid and irradiated with laser beam (PCL-LA vs. PCL-LA-IR).

##  Discussion

Resting macrophages present in tissues provide a convenient model for assessing the influence of physical and biologically active factors on their activation, which is significant in the development of new therapeutic strategies in regenerative medicine. These cells represent the baseline state from which macrophages undergo further activation and polarisation in response to infection, tissue injury, biomaterial implantation or inflammatory stimuli. Importantly, resting macrophages are one of the first immune cells to interact with biomaterials, initiating either adaptive or proinflammatory responses. For this reason, the present study applied a resting macrophage model to assess whether the simultaneous exposure to low-level laser therapy (LLLT) and α-lipoic acid (LA) released from PCL membranes can modulate macrophage function at this critical early stage of immune response.

The results of our study demonstrated that exposure to LLLT did not affect macrophage viability or adenylate kinase (AK) release, confirming the lack of cytotoxic effects of laser irradiation on macrophages cultured on a PCL substrate. Simultaneously, a marked reduction in MCP-1/CCL-2 chemokine secretion was observed in the final stage of culture, suggesting that LLLT reduces macrophage readiness to initiate a proinflammatory and chemotactic response. These observations are consistent with our previous reports indicating that appropriately selected LLLT parameters allow controlled modulation of macrophage activity without inducing excessive inflammatory signalling^22^.

This effect coincided with a pronounced decrease in MMP-2 activity at the beginning of the culture and a gradual increase of the pro-MMP-9 form over time. Among the MMP forms, transient but clearly enhanced activity of the active MMP-9 form was also observed, followed by a decrease, while the dimeric form showed a more pronounced increase in the final period of the culture. The observed changes in MMP activity may be associated with extracellular matrix remodelling processes and the regulation of cell migration, which play an important role in tissue repair^48,49^. However, this issue requires further investigation using dedicated migration/ chemotaxis assays to better elucidate the functional implications of the observed changes.

In parallel, a transient but distinct decrease in both oxidative and antioxidant potential of the cells was observed. The reduced oxidative activity may reflect a limited ability of macrophages to generate a proinflammatory response, in which reactive oxygen species exacerbate inflammation^50,51^. The concomitant reduction in antioxidant potential suggests that compensatory antioxidant mechanisms were not fully activated. This specific redox profile, characterised by a simultaneous decrease in both parameters, may favour a shift towards conditions more conducive to tissue regeneration, although further studies are required to fully elucidate its biological significance.

To date, there is a lack of studies addressing the effects of LLLT on resting macrophages cultured on PCL membranes. However, the biological effects of combining LLLT with biomaterial substrates have been described in other cellular models, clearly demonstrating that cellular responses depend on both irradiation parameters and the physicochemical properties of the material used. Karimi et al. reported increased viability of human gingival fibroblasts cultured on collagen membranes following irradiation at 915 nm and a dose of 4 J/cm²^52^. Similarly, Amid et al. demonstrated that LLLT (808 nm, 3 and 5 J/cm²) significantly enhanced the viability of human dental pulp stem cells cultured on sandblasted titanium discs, with the maximum effect observed after four days^53^. In contrast, Renno et al. showed that exposure of olfactory ensheathing cells to laser radiation (830 nm, 10 J/cm²) resulted in biomaterial-dependent effects, with increased proliferation on collagen scaffolds and a marked decrease on bioglass substrates^54^. These findings support the rationale for evaluating LLLT effects in the context of specific biomaterial–cell interactions, as performed in the present study.

The incorporation of α-lipoic acid into PCL membranes caused a transient increase in macrophage viability accompanied by a clear reduction in AK release, confirming the cytoprotective properties of LA. These effects were correlated with a marked decrease in NO and TNF-α secretion, indicating the anti-inflammatory potential of LA in this model. The observed transient decrease in pro-MMP-9 together with a simultaneous increase in MMP-9 activity may reflect short-term activation of processes related to chemotaxis and extracellular matrix remodelling.

Available literature data regarding the effects of LA on resting macrophages remain limited, and the results presented here, constitute some of the first demonstrating its ability to modulate macrophage function in a resting state. In contrast, the effects of α-lipoic acid on macrophages previously activated by inflammatory stimuli have been described in numerous studies. For example, Chang et al. showed that RAW 264.7 macrophages stimulated with histones secreted TNF-α in a mechanism dependent on the NF-κB pathway, while the use of LA significantly limited this response by inhibiting the phosphorylation of the p65 subunit, leading to attenuated NF-κB activation and, consequently, reduced TNF-α secretion^55^. Similar observations were presented by Karabay et al., who studied RAW 264.7 macrophages activated with lipopolysaccharide (LPS) and IFN-γ and assessed the effect of LA and its indole derivatives on cells. They demonstrated that all the analysed compounds significantly inhibited NO production and inducible nitric oxide synthase (iNOS) protein expression, confirming their potential in limiting the inflammatory response associated with excessive NO production^56^. Kiemer et al. also reported that in the RAW 264.7 model activated with lipopolysaccharide, α-lipoic acid significantly inhibited the production of nitric oxide and TNF-α. This effect was associated with attenuated activation of transcription factors NF-κB and AP-1, which are key for iNOS induction and proinflammatory cytokine secretion^57^. Wang et al., in turn, studied LPS- and IFN-γ-activated macrophages previously subjected to hypoxia and exhibited that LA inhibited the secretion of proinflammatory cytokines by the cells. Additionally, LA limited ROS production, decreased mitochondrial membrane potential and inhibited activation of the HMGB1/NF-κB pathway^24^. These data indicate that LA exerts anti-inflammatory effects, regardless of the type of proinflammatory stimulus. The observation of similar changes in cells not previously exposed to inflammatory activation suggests that α-lipoic acid may also influence macrophage response in resting state.

While LLLT and LA applied separately affected individual aspects of macrophage activity, their combined application led to a more complex and time-dependent modulation of inflammatory responses, redox balance and matrix remodelling. In the early phase of exposure, improved viability and increased antioxidant potential were observed, indicating favourable cellular adaptation. In later stages, sustained reduction in MCP-1/CCL-2 secretion, accompanied by limited changes in TNF-α levels, suggested maintenance of a low proinflammatory activation state and reduced chemotactic potential. The metalloproteinase profile was characterised by a pronounced reduction in MMP-2 activity and transient but clearly elevated MMP-9 activity. In the final stage of culturing, MMP-9 activity decreased, while the activity of the MMP-9 dimer increased markedly, supporting the concept of controlled extracellular matrix remodelling rather than excessive inflammatory degradation.

The observed effects may result from the synergistic photobiomodulatory effects of LLLT and the antioxidant properties of α-lipoic acid. The mechanism of action regarding LLLT, related to energy absorption by mitochondrial cytochrome c oxidase, can lead to transient modulation of respiratory chain activity, changes in mitochondrial membrane potential and the generation of reactive oxygen species with signalling properties. This can result in the modulation of redox signalling and indirectly influence the activity of transcription factors regulating the inflammatory response, including NF-κB and Nrf2^58–60^. In turn, α-lipoic acid, as a compound capable of neutralising reactive oxygen species and regenerating other antioxidants, may stabilise the cellular redox environment^26^.

In the current work, we demonstrated that the LA release from PCL membranes is characterised by a biphasic kinetic profile, consisting of an initial, faster release phase, followed by a slower, prolonged modulatory phase. According to literature reports, the kinetic parameters of delivery systems, including the method of active substance immobilisation and its release profile, can significantly influence the course of the biological response of cells^61–63^. In our research, we used the 1% LA concentration as a formulation enabling the gradual release of α-lipoic acid from the PCL membrane to biologically relevant concentrations, capable of modulating the cell response. This formulation represented a compromise between achieving a biological effect and maintaining material stability as well as controlled release of the compound.

The release profile of LA from PCL-LA membranes shows that its concentration in the culture medium reached approximately 0.08 µg/mL after 48 h and 0.16 µg/mL after 7 days, corresponding to ~ 0.78 µM. This level is below the 10–100 µM range commonly used for direct pharmacological modulation of macrophages^64,65^. Therefore, the observed biological effects are unlikely to result from LA at a high dose alone.

Instead, the low-level, sustained release of LA is intended to provide subtle redox modulation over time, which may influence NF-κB and Nrf2 signalling. In combination with LLLT, known to modulate mitochondrial activity, ROS signalling and inflammatory pathways^7,16^, this creates a synergistic microenvironment that can guide macrophage polarisation towards a reparative M2-like phenotype. Although the precise mechanisms remain to be fully elucidated, our experimental design leverages this controlled, dual stimulus to explore potential regenerative effects, while avoiding reliance on pharmacologically high doses of LA.

Our results indicate that the simultaneous use of LLLT and α-lipoic acid released from a PCL matrix may help limit the activation of proinflammatory signalling pathways, which is reflected in the observed changes in cytokine secretion, redox balance and metalloproteinase activity. The reduced secretion of MCP-1/CCL-2, TNF-α and NO suggests decreased activity of NF-κB-dependent pathways, which plays a key role in regulating the expression of proinflammatory genes, including iNOS^66–68^. Simultaneously, modulation of redox potential may influence the activity of MAPK pathways, such as p38 and ERK1/2, engaged in both the inflammatory response and the regulation of MMP expression. Modulation of redox balance, resulting from both the mitochondrial effects of LLLT and the ability of α-lipoic acid to neutralise reactive oxygen species, may lead to attenuation of NF-κB activation and simultaneous enhancement of Nrf2-dependent cytoprotective mechanisms^26,29,69^. Such signalling profile could help to attenuate the proinflammatory response of M1 macrophages, associated with the dominant activity of the NF-κB/iNOS axis, and promote functional properties similar to the M2 macrophage phenotype, in which arginase-1 and processes related to extracellular matrix remodelling play important roles^70–72^. At the same time, the changes in MMP-2 and MMP-9 activity, the reduction of the level of inflammatory mediators and the modulation of redox potential observed by us may suggest a shift in the macrophage response towards a functional profile similar to the M2 phenotype, without direct induction of polarisation.

Our research concentrated on resting macrophages, which constitute the physiological starting point for further cell activation and polarisation and therefore, the obtained results should be interpreted as an assessment of the effect concerning the studied system on the basal level of macrophage activity, without imposed proinflammatory or alternative stimulation. On this basis, it can be assumed that most probably in classically activated macrophages (M1), the combined action of LLLT and LA may lead to a suppression of the inflammatory response, while in alternatively activated macrophages (M2), it could enhance their pro-regenerative properties, related to extracellular matrix remodelling and stabilisation of redox balance. Although macrophage polarisation was not directly analysed in this study, the obtained results suggest that the cellular response to the combined action of LLLT and α-lipoic acid may depend on macrophage phenotype, which represents an important direction for further research.

### Limitation

The applied in vitro model enabled a controlled analysis of RAW 264.7 macrophage responses to the combined effects of LLLT and α-lipoic acid released from PCL membranes, allowing for the assessment of the biological potential of the investigated system. However, it should be taken into account that the simplified nature of the in vitro model does not fully reflect the complex interactions occurring in the in vivo tissue environment. Therefore, further studies involving more advanced models, such as co-culture systems and in vivo models, could provide additional information regarding the long-term cytocompatibility and potential application of the developed solution.

## Conclusions

The simultaneous use of LLLT and α-lipoic acid released from a PCL matrix limits the proinflammatory activation of macrophages while preserving the processes associated with extracellular matrix remodelling. These results indicate that combining LLLT with α-lipoic acid is a promising research strategy for the development of biomaterials with potential immunoregulatory and pro-regenerative effects.

## Data Availability

The data that support the findings of this study are available from the corresponding author.
